# Evaluation of the telemedical health care network “SAFE BIRTH” for pregnant women at risk, premature and sick newborns and their families: study protocol of a cluster-randomized controlled stepped-wedge trial

**DOI:** 10.1186/s12913-024-10667-z

**Published:** 2024-02-14

**Authors:** Helene Hense, Josephine Mathiebe, Sven Helfer, Rick Glaubitz, Mario Rüdiger, Cahit Birdir, Jochen Schmitt, Gabriele Müller

**Affiliations:** 1 Center for Evidence-Based Healthcare, University Hospital and Faculty of Medicine Carl Gustav Carus, Technische Universität Dresden, Dresden, Germany; 2 Saxony Center for Feto/Neonatal Health, University Hospital and Faculty of Medicine Carl Gustav Carus, Technische Universität Dresden, Dresden, Germany

**Keywords:** Perinatal care, Telemedicine, Stepped-wedge, Evaluation, Health services research, Implementation, Non-metropolitan regions, Study protocol

## Abstract

**Background:**

The Perinatal Center of the University Hospital Carl Gustav Carus Dresden has initiated the telemedical healthcare network “SAFE BIRTH” to coordinate and improve specialized care in non-metropolitan regions for pregnant women and newborns. The network incorporates five intervention bundles (IB): (1) Multi-professional, inter-disciplinary prenatal care plan; (2) Neonatal resuscitation; (3) Neonatal antibiotic stewardship; (4) Inter-facility transfer of premature and sick newborns; (5) Psycho-social support for parents. We evaluate if the network improves care close to home for pregnant women, premature and sick newborns.

**Methods:**

To evaluate the complex healthcare intervention “SAFE BIRTH” we will conduct a cluster-randomized controlled stepped-wedge trial in five prenatal medical outpatient offices and eight non-metropolitan hospitals in Saxony, Germany. The offices and hospitals will be randomly allocated to five respectively eight sequential steps over a 30-month period to implement the telemedical IB. We define one specific primary process outcome for each IB (for instance IB#1: “*Proportion of patients with inclusion criterion IB#1 who have a prenatal care plan and psychosocial counseling within one week”*). We estimated a separate multilevel logistic regression model for each primary process outcome using the intervention status as a regressor (control or intervention group). Across all IB, a total of 1,541 and 1,417 pregnant women or newborns need to be included in the intervention and control group, respectively, for a power above 80% for small to medium intervention effects for all five hypothesis tests. Additionally, we will assess job satisfaction and sense of safety of health professionals caring for newborns (questionnaire survey) and we will assess families’ satisfaction, resilience, quality of life and depressive, anxiety and stress symptoms (questionnaire surveys). We will also evaluate the cost-effectiveness of ”SAFE BIRTH” (statutory health insurance routine data, process data) and barriers to its implementation (semi-structured interviews). We use multilevel regression models adjusting for relevant confounders (e.g. socioeconomic status, age, place of residence), as well as descriptive analyses and qualitative content analyses.

**Discussion:**

If the telemedical healthcare network “SAFE BIRTH” proves to be effective and cost-efficient, strategies for its translation into routine care should be developed.

**Trial registration:**

German clinical trials register. DRKS-ID: DRKS00031482.

## Background

Demographic as well as structural changes in the hospital landscape challenge medical care in many regions of Germany. One of the measures taken to meet these challenges is increasing centralization of specialized care, as successfully done in other countries [1]. Centralization of care requires functioning networks between health professionals in different areas and of different disciplines. Telemedicine can connect health professionals and support the provision of specialized care in non-metropolitan regions. In the field of perinatal care, telemedicine is a feasible method of increasing access to expert care, improving parental and caregiver education, reducing transports and improving quality of care [2].

In Saxony, a federal state of Germany, the Perinatal Center of the University Hospital Carl Gustav Carus Dresden (PC-UKD) has initiated the health care network “SAFE BIRTH” (HCNSB, German: “Versorgungsnetz Sichere Geburt”) together with the largest statutory health insurance fund in this region (AOK Plus) to coordinate and improve specialized care in non-metropolitan regions of Saxony. Recent studies show the need and present structural requirements for telemedical support in perinatal care in this region [3, 4]. The telemedical network HCNSB consists of five intervention bundles:

- #1: Multi-professional, inter-disciplinary prenatal care plan for pregnant women and their unborn child.
- #2: Neonatal resuscitation.
- #3: Neonatal antibiotic stewardship.
- #4: Inter-facility transfer of premature and sick newborns.
- #5: Psycho-social support for parents.

The complex healthcare intervention HCNSB is funded by the Innovation Fund of the Federal Joint Committee (German: Gemeinsamer Bundesausschuss (G-BA)). The G-BA specifies which services in medical care are reimbursed of the statutory health insurance funds (routine care) for more than 73 million insured individuals. New forms of care funded by the G-BA have to show potential for permanent implementation into routine care. The accompanying scientific evaluation examines the effectiveness of the intervention bundles of HCNSB within the existing care structures. The results of the evaluation will play a leading role in the decision which innovations will be reimbursed of the statutory insurance funds.

The evaluation of the HCNSB examines the following hypotheses and qualitative research questions:

Primary hypothesis: The intervention bundles (IB) of the HCNSB improve care close to home compared to current routine care.

Secondary hypotheses:

1. The IB increase job satisfaction and the feeling of safety of health professionals caring for premature and sick newborns. IB furthermore increase patients’ and families’ satisfaction with care, resilience and quality of life and reduce depressive, anxiety and stress symptoms.
2. The IB are cost-effective from a statutory health insurance (SHI) perspective.

Qualitative research questions:

1. Which barriers hinder health professionals, pregnant women and families of newborns to use the IB?
2. How do the IB influence the satisfaction and sense of safety of health professionals? How do they influence the patients’ sense of safety?

A cluster-randomized controlled stepped-wedge design (SW-cRCT) was chosen to investigate the hypotheses and research questions. Randomization will be performed at the hospital respectively outpatient office level.

## Methods

The method chapter is based on the SPIRIT reporting guidelines [5].

**Fig. 1:**
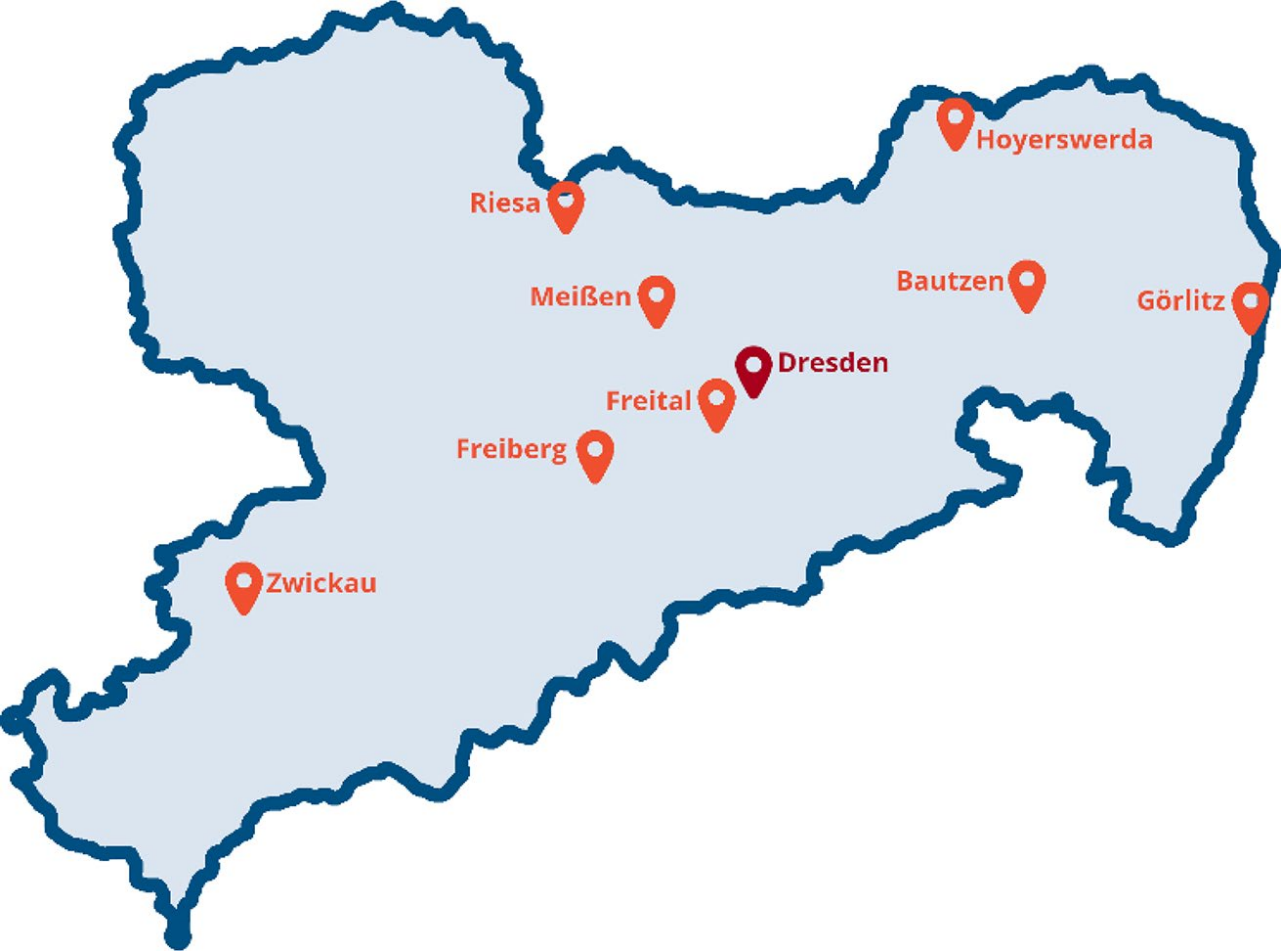
Locations of study sites of the HCNSB in Saxony (Dresden as location of the coordinating facility in red)

The Perinatal Center of the University Hospital Carl Gustav Carus Dresden (PC-UKD) coordinates the HCNSB. Five prenatal medical outpatient offices and eight hospitals participate in the project (see Fig. 1). They are members of the Saxony Center for Feto/Neonatal Health. All study sites are listed in the trial registration (DRKS00031482).

### Eligibility criteria

All patients or newborns meeting the following criteria will be included:

- IB#1: Pregnant women with a disorder affecting fetal health (e.g. gestational diabetes) or with a suspicion of a serious disease of the fetus who are cared for on an outpatient basis by a participating prenatal obstetrician,
- IB#2: Newborns cared for in the delivery room or admitted as inpatients who required resuscitation or intensive care at the participating hospital,
- IB#3: Newborns admitted as inpatients who receive antibiotic therapy at the participating hospital and/or have a positive microbiologic screening result,
- IB#4: Premature or sick newborn receiving inpatient care at a participating hospital or the PC-UKD with a suspected need for transfer to the PC-UKD or indication for transfer back close to home,
- IB#5: Families of newborns hospitalized at a participating hospital for more than 5 days.

The secondary study population consists of outpatient obstetricians and health professionals at the hospitals using IB.

### Intervention bundles (IB) of the health care network “SAFE BIRTH” (HCNSB)

#### Intervention bundle #1: Multi-professional, inter-disciplinary prenatal care plan

In Germany, midwives and outpatient obstetricians care for pregnant women. In the event of any abnormalities, pregnant women are examined by a specialist for prenatal diagnostics. If the suspicion of a fetal abnormality or maternal risk is confirmed, prompt multi-disciplinary and inter-professional planning of further steps is necessary. Currently, the development of an individual care plan is usually done in a specialized perinatal center. This current process is associated with lengthy waiting times and increased health care costs [3]. Psycho-social aspects are often not adequately addressed [3].

IB#1 is aimed at all pregnant women with fetal or maternal abnormalities requiring control at the PC-UKD and consists of the following measures:

- The **prenatal tele-consultation** enables the outpatient obstetrician to have an immediate virtual consultation with the expert of the PC-UKD while the pregnant woman is still present.
- In the **prenatal jour-fixe**, recorded findings are discussed in the virtual room, without the participation of the pregnant woman.
- Subsequently, the **prenatal care plan** is determined, if necessary with the involvement of other disciplines.
- In addition, the psycho-social team of the PC-UKD contacts the pregnant woman by telephone, thus ensuring prompt **psycho-social counseling** tailored to the individual characteristics.
- A virtual **feto-neonatal board meeting** serves to review and adapt the prenatal care plan to current findings during care.

#### Intervention bundle #2: Neonatal resuscitation

Neonatal resuscitation is a rare event. However, immediate and adequate care of infants is a prerequisite to prevent long-term harm. Specialized neonatal expertise is not available at all facilities, and it can take up to an hour for a neonatal expert from a NICU to arrive on scene. For this reason, it is important that on-site pediatricians have the immediate ability to access the neonatal experts’ support via telemedicine. In some cases, this may even eliminate the need for a neonatologist to travel to the hospital or to transfer the newborn [3].

IB#2 is specifically designed to ensure **immediate availability of neonatal expertise** during neonatal resuscitation. The participating hospital receives **telemedical support** from the neonatology department of the PC-UKD directly after the call. If needed, a neonatal transport can be arranged in parallel and the neonatologist on route can stay informed about the current status of the resuscitation via push messages. In the follow-up of the supported resuscitation, a structured debriefing takes place to optimize the cooperation and to enable quality assurance of IB#2.

The goal is to **reduce neonatal transports** and **improve neonatal care** in critical situations.

#### Intervention bundle #3: Neonatal antibiotic stewardship

Antibiotics are among the most commonly used medications in neonatal units due to the high morbidity and mortality due to neonatal infections. Antibiotic stewardship has been shown to improve rational antibiotic use [6], but the specific expertise in neonatal infectious disease is not available in every institution.

IB#3 ensures appropriate consultation with a pediatric infectiologist at the PC-UKD. If antibiotic treatment is started for a newborn at a participating hospital or if an abnormal microbiological finding is detected, a telemedical infectiology consultation is conducted within 48 h with the aim of recommending an individualized antibiotic therapy.

This is intended **to avoid overtreatment** when antibiotic therapy has already begun or in advance.

#### Intervention bundle #4: Inter-facility transfer of premature and sick newborns

Once the child is stable and no longer needs the highly specialized neonatal care, it is the aim to transfer them close to home. However, this requires a continuous trans-institutional care and that the parents are appropriately prepared [4].

IB#4 provides support to participating hospitals via neonatal tele-consultations and as part of remote neonatal jour fixes. Through the regular exchange of information about neonates who are eligible for transfer close to home, **transfers from the PC-UKD to the hospitals will be promoted and transfers to the PC-UKD will be reduced**. The transfer process to the participating hospitals is accompanied by psycho-social support services and the parents are involved in these joint “remote rounds” in order to reduce uncertainties and enable an optimal flow of information. The jour fixes also serve as quality assurance measure by debriefing children who have already been transferred.

#### Intervention bundle #5: Psycho-social support for parents

The aim of professional psycho-social support in neonatology is to improve parent-child bonding and to optimize medical treatment by helping to organize follow-up and preventive care appointments. The positive effects of these psycho-social support services have been published several times [7, 8]. However, development and implementation of recommended measures require substantial financial, organizational and personnel resources. Notably, the highly specialized staff needed is often not available for the time-consuming counseling work in smaller clinics.

In IB#5, families who stay more than 5 days in the participating hospital receive the offer of telemedical support based on the psycho-social expertise of the PC-UKD. Families are offered virtual parent training, telephone counseling sessions and support in contacting local psycho-social services.

**Fig. 2:**
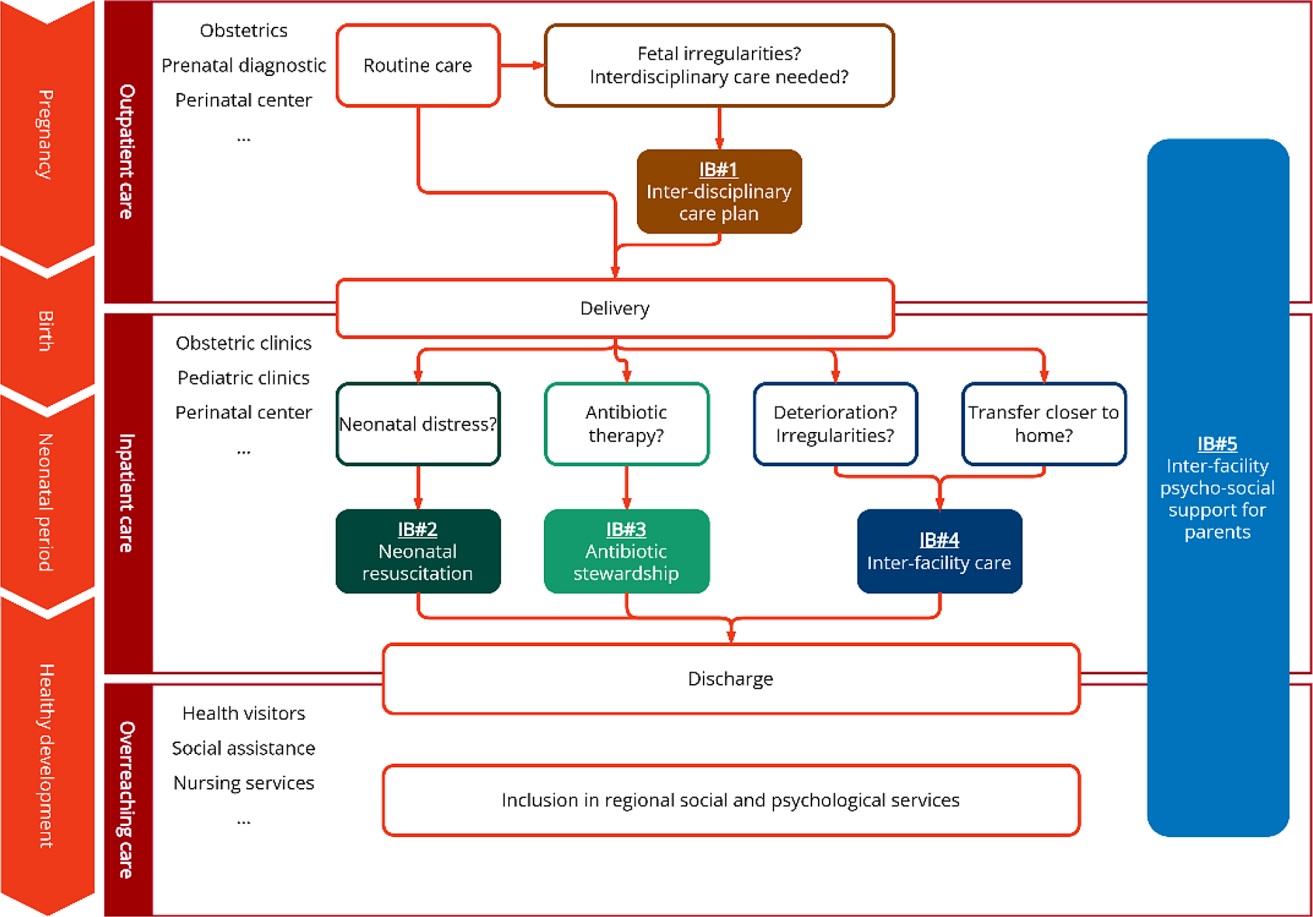
The five intervention bundles (IB) of HCNSB

Figure 2 provides an overview of all five IB of the HCNSB.

### Technical implementation of the intervention bundles (IB)

Eckart et al. [4] describe the details of the basic technical implementation. The project coordinator decided to use the JOIN messenger application (Allm Inc., Tokyo, Japan), which is certified as a medical device. Special attention was paid to pseudonymized, GDPR compliant data use during implementation. It turned out that the implementation by means of mobile devices is the most suitable for most IB, since the audio and video capabilities of modern devices are sufficient for this. The JOIN web application seems most appropriate for IB#1, where ultrasound images or videos are transmitted via HDMI converter, and in other IB when additional devices are necessary, for example for larger conferences.

### Outcomes

The outcomes refer to the interventions of the intervention bundles IB#1 to IB#5. Primary process outcomes are (data sources in brackets are described in more detail in chapter “Data management and collection”):

- IB#1: Proportion of patients with inclusion criterion IB#1 who have a prenatal care plan with a multi-professional recommendation for postnatal care and psychosocial counseling within one week (data source: project database),
- IB#2: Proportion of neonates with inclusion criterion IB#2 for whom neonatology expertise is available in the delivery room < 15 min postnatally (including telemedicine, if applicable) (data source: project database),
- IB#3: Proportion of newborns with inclusion criterion IB#3 in which antibiotic could be discontinued within 48 h (in case of CrP, IL-6 negative and negative blood culture) or antibiotic therapy for 6 days (in case of pos. blood culture) with antibiotic was performed according to resistogram (data source: project database),
- IB#4: Proportion of newborns cared for at the UKD from the region (according to zip code list) who were finally discharged from a hospital close to home (data source: project database),
- IB#5: Proportion of patients with inclusion criterion IB#5 who received at least one psychosocial support service (data source: project database).

Secondary outcomes are listed in Table 1.

**Table 1:** Secondary outcomes and related data sources

Intervention bundle	Secondary outcome	Data source
**Care close to home**		
1	Proportion of patients referred to the Perinatal Center of the University Hospital	SHI routine data of the participating health insurance company
	Proportion of patients who gave birth in a defined facility level	project database, SHI routine data of the participating health insurance company
	Proportion of patients and mean number of virtual feto-neonatal board meetings	project database
	Frequency of disciplines involved in virtual feto-neonatal board meetings	project database
	Proportion of patients with psychosocial consultation within two weeks after diagnosis or directly at patient contact	project database
	Proportion of patients who make use of further offers of help	project database
	Maternal risks and complications during inpatient stay measured by ICD and OPS codes	SHI routine data of the participating health insurance company
2	Number of postnatal transports	project database
	Proportion of transferred newborns	project database
	On transport: time of neonatologist at participating hospital	project database
	Condition of newborn during transport measured by body temperature and blood glucose in each case in participating hospital and Perinatal Center of the University Hospital	project database
	Time (in minutes) until a neonatologist is present in the delivery room postnatally (also virtually, if necessary)	project database
	Proportion of infants with hypoglycemia in the first 24 h	project database
	Number of teleconsultations in the delivery room	project database
	Proportion of newborns with teleconsultation in case of deterioration of condition on ward	project database
	Number of deceased newborns	project database, SHI routine data of the participating health insurance company
3	Proportion of newborns with antibiotic therapy	project database
	Average duration of antibiotic therapy	project database
	Proportion of newborns whose therapy was terminated after 36–72 h	project database
	Proportion of newborns with pathogen-specific adjustment of therapy after pathogen identification	project database
	Proportion of newborns with use of reserve antibiotics	project database
	Proportion of reserve antibiotics in total antibiotic consumption	project database
	Proportion of newborns with adequately performed pharmacologic surveillance	project database
4	Length of stay of newborns at the Perinatal Center of the University Hospital originating from the participating hospitals	project database, SHI routine data of the participating health insurance company
	Total duration of inpatient care of newborns from participating hospitals	project database, SHI routine data of the participating health insurance company
	Proportion of newborns who had to be transferred back to the Perinatal Center of the University Hospital after transfer to participating hospital	project database, SHI routine data of the participating health insurance company
5	Proportion of patients for whom the previously individually assessed need for follow-up care and networking was achieved	project database
	Proportion of guardians with offers of help (conversations/guidance by staff of the clinic providing care, consultations with psychiatry or psychosomatics of the clinic providing care, referral to other offers of help (e.g. youth welfare office, family midwife, home help, etc.) by clinic providing care, help refused, no need for help seen on the part of the clinic providing care)	project database
	Proportion of guardians who used IB#5 measures	project database
**Satisfaction and sense of safety of participating health professionals in the care of pregnant women and newborns**		
1–5	Telemedicine contacts with the university hospital - helpful or not and reasons why	telemedicine questionnaire survey
	Can the questions be answered via telemedicine	telemedicine questionnaire survey
	Job satisfaction	questionnaire survey, self-developed items based on COPSOQ (Kristensen et al., 2005; German version Nübling et al., 2006)
	Sense of safety in neonatal care	questionnaire survey, self-developed case vignettes
	When / why do health professionals in the respective participating facilities use the intervention bundle and when / why not?	questionnaire survey, self-developed items; qualitative interviews
	What has changed as a result of the intervention bundle - both positively and negatively?	qualitative interviews
	Reasons for (dis)satisfaction	qualitative interviews
	Reasons for feeling safe	qualitative interviews
**Satisfaction with medical care and resilience of patients and families (questionnaire survey 3 months after study inclusion for IB#1 and 3 months after calculated delivery date for IB#2–5; qualitative interviews with selected individuals)**		
1–5	Satisfaction with care	self-developed items
	Reasons for (dis)satisfaction	qualitative interviews
	Resilience	BRS - Brief Resilience Scale (Chmitorz et al., 2018)
	Reasons for resilience	qualitative interviews
	Quality of life	WHOQOL-BREF (The WHOQOL Group, 1998)
	Depressive, anxiety and stress symptoms	DASS-P: Depression Anxiety Stress Scale for the Peripartal Period (Martini et al., 2009)
	Reasons for use of individual services	qualitative interviews
**Economic impact**		
1	Number of duplicate clinic presentations: Referrals to the Perinatal Center of the University Hospital	project database
	Required travel distances to the medical examination at the Perinatal Center of the University Hospital: travel distance from the patient’s home to the university hospital and back	project database
	Inpatient costs of mother (for 6 months before birth)	SHI routine data of the participating health insurance company
	Number of transfers of the mother	SHI routine data of the participating health insurance company
	Costs of transports between hospitals	SHI routine data of the participating health insurance company
	Duration of inpatient stays of the mother (for 6 months before birth)	SHI routine data of the participating health insurance company
	Outpatient costs for gynecological specialists and prenatal physicians	SHI routine data of the participating health insurance company
	Periods of inability to work during pregnancy	SHI routine data of the participating health insurance company
	Expenditures during the inpatient stay of the mother (measured by OPS codes)	SHI routine data of the participating health insurance company
2–5	Inpatient costs of the child (up to 6 months after birth)	SHI routine data of the participating health insurance company
	Number of transfers of the child related to the inpatient stay at birth	SHI routine data of the participating health insurance company
	Costs of transports between hospitals	SHI routine data of the participating health insurance company
	Duration of ventilation time [hours] of the child	SHI routine data of the participating health insurance company
	Number of inpatient stays of the child (for the 6 months after first discharge after birth)	SHI routine data of the participating health insurance company
	Expenditures for the child during the inpatient stay (measured by OPS codes)	SHI routine data of the participating health insurance company
**Expenditures for the novel form of care**		
1–5	Type, duration, number and type of participants are recorded for each measure	project database

### Sample size and recruitment

The first patient/newborn was included on 29 January 2023. The inclusion of the last patients and newborns will take place by 30 June 2025 at the latest.

We estimated a separate multilevel logistic regression model for each primary outcome. Across all IB, 1,417 pregnant women or newborns need to be included in the control group and 1,541 in the intervention group. Calculations are based on consortium leadership surveys from April 2020 to April 2021 among participating health professionals [3] and yielded power above 80% for small to medium intervention effects for all five hypothesis tests. The intervention effect results from the change in the probability of achieving the target between the intervention and control phases. A drop-out rate of 10% was calculated.

Participating prenatal medical outpatient offices and hospitals recruit the study participants.

### Assignment of interventions

**Fig. 3:**
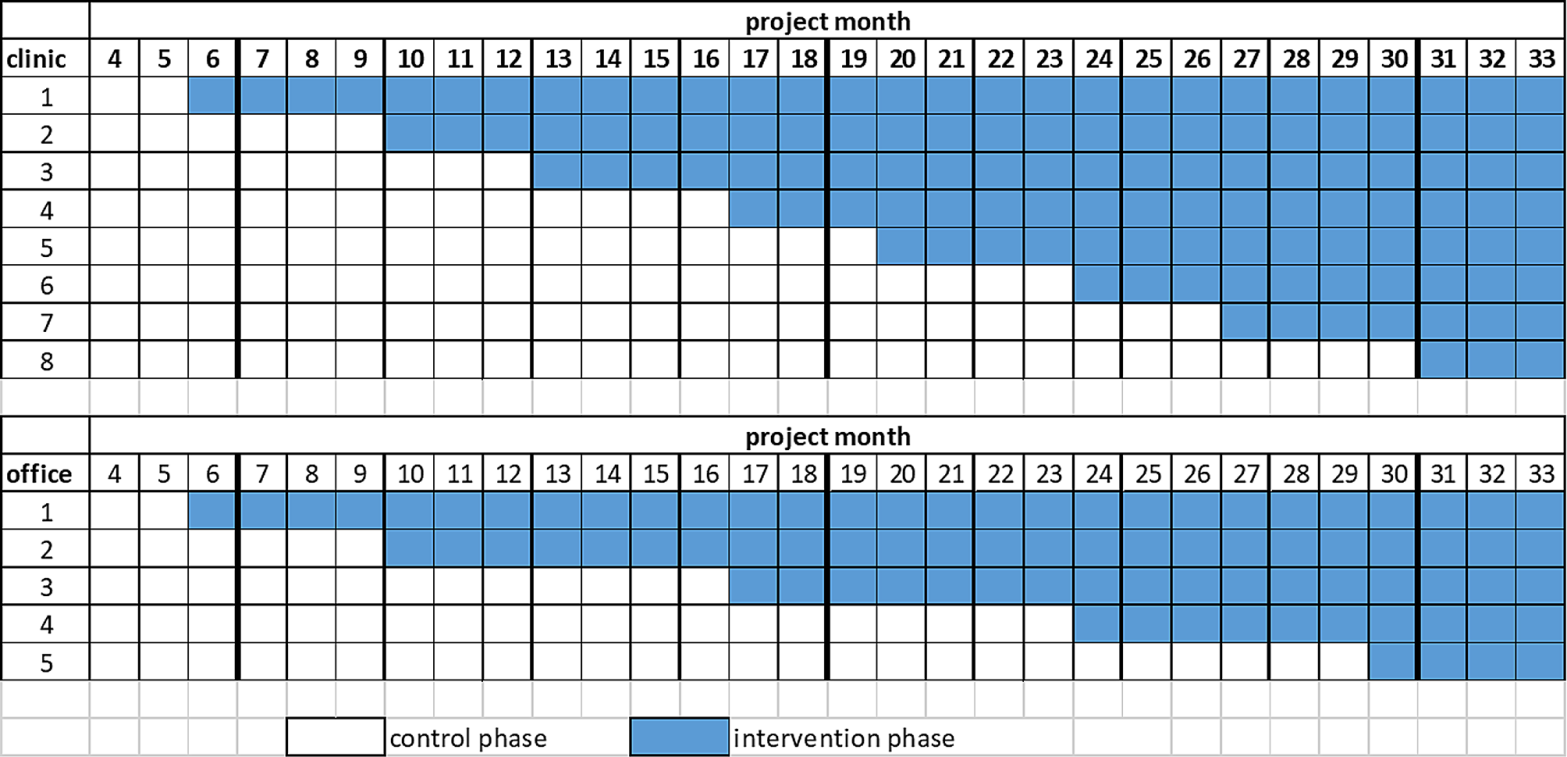
Result of the sequence generation for clinics and outpatient offices

The decision whether a newborn/patient is assigned to the intervention or control group depends on the time of inclusion. If the recruiting facility is in the control phase, the patient or newborn is assigned to the control group, otherwise to the intervention group. There is no blinding. The order in which the recruiting facilities enter the intervention phase is randomized. Figure 3 illustrates the allocation of clinics and prenatal medical outpatient offices to the intervention time points defined within the 30-month observation period.

### Data collection and management

**Fig. 4:**
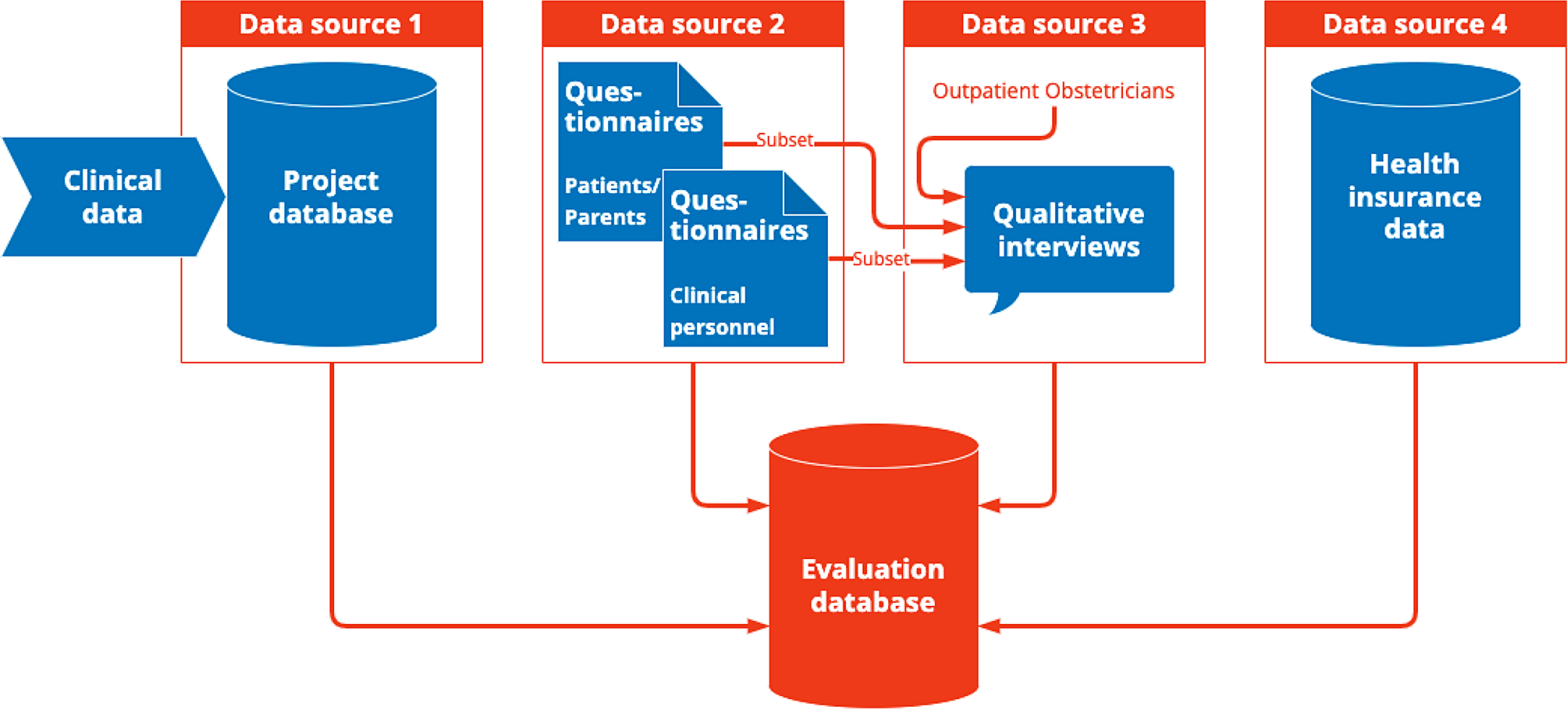
Data sources

The primary and secondary outcomes are collected via three prospective primary data sources and one secondary data source (Fig. 4). Data source (1) is a project database (REDCap), in which patient cases and process data are documented. For instance, data on the health status of the pregnant woman and the newborn, referrals and interventions carried out are recorded, and process data on the type, duration, number and type of participants per intervention. Data source (2) is composed of two questionnaire surveys. Patients or parents and guardians of newborns are given access to an online questionnaire 3 months after study inclusion, which measures their satisfaction with care (self-developed), resilience (BRS - brief resilience scale [9]), quality of life (WHOQOL-BREF [10], and psychosocial distress (DASS-P - Depression Anxiety Stress Scale for the Perinatal Period [11]). The second questionnaire survey is designed for health professionals at participating hospitals caring for newborns. They will receive a paper questionnaire at 3 measurement points, which measures their sense of safety in the care of newborns by means of self-developed case vignettes and job satisfaction (based on COPSOQ (Copenhagen Psychosocial Questionnaire) [12], German version [13]) in the course of the project. Semi-structured individual telephone interviews will be the data source (3). Qualitative interviews will be conducted with 5 outpatient obstetricians, 12 inpatient health professionals and 12 patients and guardians of newborns about barriers to the use of IB and reasons for the quantitatively measured outcomes. Routine SHI data of the participating health insurance companies will be the data source (4). Among other things, referrals, transports and transfers, complications, inpatient length of stay, travel distances and costs will be used as the data basis for the evaluation.

The German association “Das Frühgeborene Kind e. V.” (English: “The preterm child”, a registered association to support preterm children and their parents by education, information, offers of help, research and political participation) was involved in the development of the questionnaire set for patients and guardians. The representatives of the association received a proposal for the outcome dimensions recorded in the questionnaire set and had the opportunity to add additional outcomes. At the same time, they received a draft questionnaire with a request for critical review and the opportunity to add further ideas. As the feedback from the representatives was transferred to the questionnaire set and to the web application SoSci Survey [14], three members of the study council of the association tested the questionnaire set for comprehensibility and feasibility. Based on these pre-tests, we were able to revise unclear questions. In addition, we added justifications for the use of standardized instruments to the questionnaire to increase compliance among respondents.

Qualitative sampling plans will be developed for the selection of heterogeneous interviewees. In terms of a mixed methods design, the results from the quantitative questionnaire survey also serve this selection. If possible, the interviewees should be heterogeneous regarding their satisfaction and sense of safety in order to explore why some individuals benefit more and others less from the intervention and whether the effects are due to the intervention or also to other accompanying circumstances. This is an explanatory sequential design [15] because the qualitative survey builds on the quantitative survey.

Data management procedures are fixed in a data protection concept. Details of data management procedures can be requested from the authors.

### Data analysis

As part of the confirmatory analysis, we examine the effectiveness of the intervention bundles using the IB-specific primary outcomes (Chap. 2). A separate logistic multilevel regression model is estimated for each primary outcome, which contains the intervention status (control or intervention phase) of the respective patient or newborn facility as an explanatory variable. The coefficient of this explanatory variable represents the intervention effect. To account for the clustered structure and the possible correlation of patient outcomes within the institutions, all models contain a random intercept at the institutional level [16]. The estimation results support the respective primary hypothesis if the estimated intervention effect is positive and statistically significant. Significance is tested using a two-tailed test with a significance level of 5%. To control for the inflation of the Type 1 error in our setting where we conduct multiple tests, Holm-Bonfferoni correction is used [17].

For testing of hypothesis 1, we compare data for the control and intervention group by analyzing frequencies and mean values, confidence intervals and conducting adjusted regression models.

The qualitative semi-structured interviews will be audio recorded and transcribed verbatim. The transcripts will be analyzed content-analytical using inductive and deductive coding.

Secondary hypothesis 2 (health economic evaluation) will be assessed by analyzing statutory health insurance routine data for each patient case. For each intervention bundle, we sum up the inpatient costs and costs for transfers of the individual patients, and, if mother and child are insured by AOK Plus, costs associated to their newborns in the context of their birth and costs in the 6 months following birth. In the next step, we compare average costs between the intervention and control phase cases and determine the difference in costs. Additionally, we compare the following aspects between the control and intervention group: patient transfers; length of inpatient stays; risks, complications and expenses during the inpatient stay; and more frequent clinic presentations. Expenses for patients and the medical staff involved will be included in the analysis (Table 1, last line).

### Monitoring

The project coordination records the data centrally, documents problems as well as implausible data and clarifies them in monthly meetings with the recruiting offices and hospitals. At least once a year, the project coordination visits the recruiting facilities to monitor the recruitment process.

We do not expect any adverse events, because intervention patients receive routine care and the telemedicine in addition. There is a low risk of data misuse. The continuous exchange with the recruiting facilities and the multi-method approach, including qualitative interviews with patients and medical staff, aims to uncover potential unintended effects.

No audits are planned.

## Discussion

For the HCNSB, the stepped-wedge design offers the advantage of implementing the intervention at the outpatient offices and hospitals at subsequent time points. Compared to classic cluster-randomized studies, the SW-cRCT design offers the advantage that the intervention does not have to be withheld from any participating institution during the study phase. Furthermore, SW-cRCTs prove to be superior to classic cluster-RCTs in terms of power, particularly in the case of a high intra-cluster correlation. However, the design also comes with limitations [18, 19].

Measurement of secondary outcomes via the questionnaire surveys will be across IB and not IB-specific. Qualitative interviews will additionally explore how IB affect the outcomes measured and what barriers to the use of all IB exist on the part of health professionals, pregnant women, and families. They provide supporting information why some individuals benefit more and others less from certain IB and to which individual interventions this is attributable.

Due to the limited project duration, the project is not preceded by a pilot phase. In the start-up phase, adjustments were made to the documentation forms. This was done before the first office and hospital started the intervention phase.

The project started in October 2022. We aim to finish our analyses until spring 2026. Therefore, publication of the results can be expected at the end of 2026 or at the start of 2027.

## Data Availability

No datasets were generated or analysed during the current study.

## Abbreviations

HCNSB: health care network “SAFE BIRTH” (German:“Versorgungsnetz Sichere Geburt”)
IB: intervention bundle(s)
PC-UKD: Perinatal Center of the University Hospital Carl Gustav Carus Dresden
RCT: Randomized controlled trial
SW-cRCT: Stepped wedge cluster randomized controlled trial
